# Supplementary material for: The Emergence of SARS-CoV-2 Variant(s) and Its Impact on the Prevalence of COVID-19 Cases in the Nabatieh Region, Lebanon
Source: Med Sci (Basel). 2021 Jun 2;9(2):40. doi: 10.3390/medsci9020040 (PMC8293406; doi:10.3390/medsci9020040)
Supplement: Supplementary file 1 [file medsci-09-00040-s001.zip › medsci-1193603-supplementary.pdf]

Supplementary Material:

Table S1. Nucleic acid extraction kits mostly used in the study.

Nucleic acid extraction kit	Manufacturer	Utilization	Technology
QIAamp® Viral RNA Mini Kit	Qiagen, Germany	Manual	Spin columns
AccuPrep® Viral RNA Extraction Kit	Bioneer, Korea	Manual	Spin columns
MagMAX™ Viral/Pathogen II (MVP II) Nucleic Acid Isolation Kit	Thermo Fisher Scientific, USA	Automated	Magnetic beads
ID Gene™ Mag Virus Extraction Kit	ID-Solutions, France	Automated	Magnetic beads

Table S2. COVID-19 RT-PCR kits mostly used in the study.

COVID-19 RT-PCR Kit	Manufacturer	Gene Targets
TaqPath™ COVID-19 CE-IVD RT-PCR Kit	Thermo Fisher Scientific, USA	*S* gene<br>*N* gene<br>*ORF1ab* gene
Maccura SARS-CoV-2 Nucleic Acid Detection Kit	Maccura Biotechnology, China	*E* gene<br>*N* gene<br>*ORF1ab* gene
GeneFinder™ COVID-19 Plus RealAmp Kit	GeneFinder; Infopia Inc., Anyang, Korea	*E* gene<br>*N* gene<br>*RdRp* gene

Table S3. Results interpretation.

SARS-CoV-2 *ORF1ab*	SARS-CoV-2 *N*	SARS-CoV-2 *S*	Result
Negative	Negative	Negative	SARS-CoV-2 not detected
	Only one SARS-CoV-2 target positive		SARS-CoV-2 inconclusive
	Two or more SARS-CoV-2 targets positive		SARS-CoV-2 positive
